# Molecular mutations as a possible factor for determining extent of thyroid surgery

**DOI:** 10.1186/s40463-019-0372-5

**Published:** 2019-10-17

**Authors:** Joshua R. Krasner, Nourah Alyouha, Marc Pusztaszeri, Veronique-Isabelle Forest, Michael P. Hier, Galit Avior, Richard J. Payne

**Affiliations:** 1 Faculty of Science, McGill University, 853 Sherbrooke Street West, Montreal, QC Canada; 2 Department of Otolaryngology Head and Neck Surgery, Sir Mortimer B. Davis-Jewish General Hospital, McGill University, Montreal, QC Canada; 3 Department of Pathology, Sir Mortimer B. Davis-Jewish General Hospital, McGill University, Montreal, QC Canada; 4 Department of Otolaryngology Head and Neck Surgery, Technion, Faculty of Medicine, Hillel-Yaffe Medical Center, Hadera, Israel

**Keywords:** Thyroid cancer, Molecular testing, Extent of surgery, *BRAF V600E*, *TERT*, *RAS*

## Abstract

**Background:**

Molecular testing of thyroid nodules is a diagnostic tool used to better understand the nature of thyroid nodules. The aim of this study is to better comprehend the relationship between specific mutations and aggressive behavior of the tumour as demonstrated on postoperative pathological analysis.

**Methods:**

A retrospective chart review of 103 cases was performed. Included were patients who had undergone molecular testing using a panel that tests for 9 mutations (ThyGenX®) and were found to have malignant tumours. The following gene alterations were found pre-operatively in the nodules: *BRAF V600E* (*n* = 32), *BRAF K601E* (*n* = 4), *NRAS* (*n* = 11), *HRAS* (*n* = 4), *KRAS* (*n* = 3), *RET/PTC1* rearrangement (*n* = 1), *TERT* promoter (*n* = 2), *PAX8-PPARγ* rearrangement (*n* = 1), and 45 cases where no mutation was detected. Aggressive behavior was defined by extra-thyroidal extension (ETE), lymph node metastasis (LN+), and the following variants of papillary thyroid carcinoma: tall cell, solid, diffuse sclerosing, columnar cell and hobnail. Chi-squared testing was performed to compare groups.

**Results:**

The group with *BRAF V600E, RET/PTC1* rearrangement, and *TERT* promoter mutations was associated with ETE 37.1%, and LN+ 45.7% of the time compared to 4.3 and 13.0% in the group with other mutations, and 4.4 and 4.4% in the group with no mutations (*p*-value 0.02, *p*-value < 0.001, p-value 0.006). In addition, the *BRAF V600E, RET/PTC1* rearrangement*,* and *TERT* mutations group demonstrated tall cell variants (17.1%), columnar cell variants (5.7%), and hobnail variants (3%). The other mutations group demonstrated columnar cell variants (4.3%), and the no mutations group demonstrated solid variants (2.2%).

**Conclusions:**

In this study, *BRAF V600E, RET/PTC1* rearrangement*,* and *TERT* mutations were associated with aggressive behaving thyroid malignancies as defined above. Molecular testing may be a useful method to anticipate aggressive tumour types and therefore assist in planning the extent and timing of surgery.

## Background

Current preoperative thyroid nodule evaluation strategies such as ultrasound (U/S) and fine needle aspiration (FNA) have been around since the 1970’s. These modalities have improved diagnostic accuracy. Nevertheless, approximately 20-25% of thyroid nodules remain indeterminate after U/S and FNA, highlighting the need for more accurate testing [1]. Molecular testing of thyroid nodules is a diagnostic tool used to better understand the nature of thyroid nodules, thereby improving patient care [2].

Studies have attributed certain tumour characteristics/behaviours with specific mutations.

The oncogenic *BRAF V600E* mutation occurs in about 40-45% of papillary thyroid cancers (PTC). It activates the MAPK signaling pathway in human cancer and has been shown to correlate with aggressive features in PTC, including extrathyroidal extension (ETE), lymph node metastasis (LN+), and the tall cell variant [3]. An increasing number of studies that include meta-analyses have been able to demonstrate an association between *BRAF V600E* status and aggressive tumour behavior [4–9]. Other studies, however, could not confirm this data, which has resulted in uncertainty about the value of *BRAF V600E* in PTC [10–15]. *BRAF V600E* mutations have also been shown to have more aggressive features on pre-operative US [16].

Activation of the telomerase reverse transcriptase (*TERT*) gene, which encodes for the catalytic subunit of telomerase, is implicated in tumorigenesis and cell immortalization. *TERT* promoter mutations were recently reported in thyroid cancer [17, 18] where they have been associated with aggressive cancers [17].

*RAS* mutations on the other hand may be more indolent with one study showing that they are related to more encapsulated tumours and lower rates of lymph node metastasis [1]. *RAS* mutations are also present in benign follicular adenomas [3].

The aim of this study was to investigate whether there is a relationship between molecular mutations tested for by ThyGenX®: *BRAF V600E*, *BRAF K601E*, *HRAS*, *NRAS*, *KRAS*, *TERT*, *PAX8-PPARγ*, *RET/PTC1*, *RET/PTC3*, *PIK3CA* and aggressive post-operative features on pathological analysis.

## Methods

### Study design

This is a retrospective chart review of 103 medical charts performed at a McGill University Teaching Hospital in Montreal, Quebec. Data on baseline characteristics, results of molecular mutation testing and postoperative pathology was collected. The specific type of malignancy (papillary thyroid carcinoma, follicular carcinoma, hurthle cell carcinoma, poorly differentiated thyroid carcinoma) and the specific variant (classical, follicular, oncocytic, tall cell, columnar cell, solid, hobnail) were recorded. Noninvasive follicular thyroid neoplasm with papillary-like nuclear features (NIFTP) tumors were recorded as well (Table 1). See Additional file 1 for all data collected. Aggressive tumours were defined as having at least one of the following features: ETE, LN+ or any of the following variants of papillary thyroid carcinoma (tall cell, solid, diffuse sclerosing, columnar cell, hobnail) as per the postoperative pathology report. This study (CR17-52) was approved by the Research Ethics Committee at the Jewish General Hospital, Montreal, Quebec.

**Table 1:** Histological types

	BRAF V600E	BRAF K601E	RAS	RET/PTC1	TERT	PAX8-PPAR_γ_	No Mutation	Total
Papillary carcinoma								
Classical	22	0	1	1	0	0	17	41
Follicular	3	4	13	0	1	1	13	35
Oncocytic	0	0	0	0	0	0	4	4
Tall cell	4	0	0	0	1	0	0	5
Columnar cell	2	0	1	0	0	0	0	3
Solid	0	0	0	0	0	0	1	1
Hobnail	1	0	0	0	0	0	0	1
Follicular carcinoma (Minimally invasive)	0	0	0	0	0	0	4	4
Hurthle cell carcinoma	0	0	0	0	0	0	1	1
NIFTP	0	0	3	0	0	0	5	8

### Patient selection

The charts of patients who underwent pre-operative molecular testing using ThyGenX® from January 2016 to February 2018 were reviewed. Patients who had surgery and confirmed thyroid malignancy of the nodule that underwent molecular testing were included in this study. Patients with benign tumours on final pathology or who had undergone molecular testing using other testing methods were excluded. Patients were divided into three groups. The first group consisted of patients that tested positive for *BRAF V600E, RET/PTC1* rearrangement*,* or *TERT* promoter mutations [19]. The second group consisted of patients that tested positive for *BRAF K601E*, *HRAS*, *NRAS*, *KRAS*, and *PAX8-PPARγ* rearrangement. The third group consisted of patients where no mutation was detected on molecular testing.

### Sample collection

After obtaining informed consent from the patient, ultrasound guided FNA was performed to collect the thyroid nodule sample for molecular testing. The specimen was then added to RNARetain® (a preservative solution), after which the sample was transported at room temperature via courier to Interpace Diagnostics in Pittsburgh, United States.

The surgical pathology specimens were reported by experienced thyroid pathologists who routinely comment on aggressive features as defined by: tumours with ETE, LN+, and the following variants of papillary thyroid cancer: tall cell, solid type, diffuse sclerosing, columnar cell, and hobnail.

### Statistical analysis

One-way ANOVA was performed to compare groups. If a statistical significance was suspected an ad-hoc analysis using Dunnet’s-T3 was performed to determine *p*-value. Significance was confirmed at a *p* value < 0.05. Relative risk calculations were performed using SPSS software.

## Results

### Baseline characteristics

A total of 103 charts were included in this study. Baseline information was calculated for each tumour. This included age, gender, the longest axis measurement in centimeters (cm) of the tumour as per the post-operative pathology report, the longest axis measurement in cm of the tumour as per the pre-operative U/S assessment, and the ultrasound guided FNA biopsy results using the Bethesda classification or interpreted Bethesda classification using cellblock technique or cytospin analysis. The baseline characteristics of each group is summarised in Table 2. No statistically significant differences were detected with regards to age and gender. Apart from *TERT* promoter mutated tumours, which were found to be statistically larger than *BRAF K601E* mutated tumours (*p* < 0.05), there were no differences with regards to tumour size.

**Table 2:** Baseline characteristics

Mutation	n	Mean age	Gender (F:M)	Mean final pathology size (cm)	Mean U/S size (cm)	FNA bethesda score distribution (%)			
						VI	V	IV	III
*BRAF V600E*	32	46.53	1.21	1.70	1.8	87	13	0	0
*BRAF K601E*	4	47.00	1.5	1.08*	1.2	0	75	25	0
*RAS*	18	45.83	1.33	2.38	2.4	28	33	11	28
*TERT*	2	67.00	1	2.80*	2.1	50	0	50	0
*RET/PTC1*	1	46.00	1	1.50	1.80	100	0	0	0
*PAX8/PPARγ*	1	51.96	0	1.00	1.40	0	0	100	0
No mutations	45	51.96	1.31	1.85	2.2	29	38	22	11

*U/S* ultrasound; *FNA* fine needle aspiration

*Statistically significant difference with regards to tumour size (*p*-value < 0.05)

**Fig. 1:**
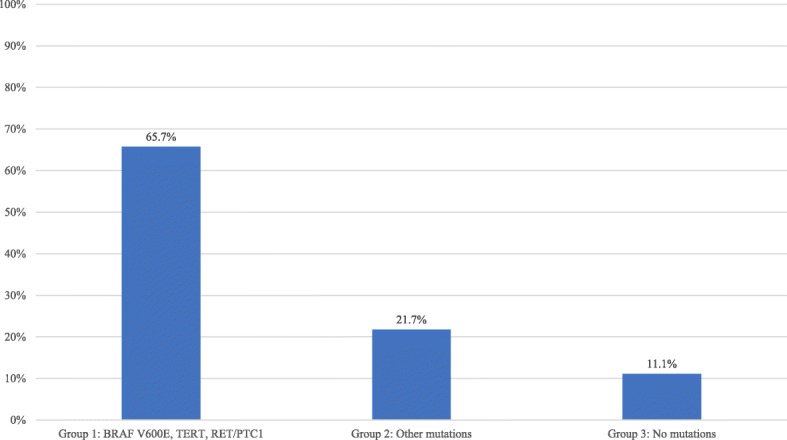
Aggressive cancers per group (%). Percentage of aggressive tumours was calculated for each group

Group 1 (*BRAF V600E, RET/PTC1,* and *TERT*) consisted of 35 nodules. Group 2 (*NRAS*, *HRAS*, *KRAS*, *BRAF K601E*, and *PAX8-PPARγ*) consisted of 23 nodules. Group 3 (no mutation detected) consisted of 45 nodules. Group 1 demonstrated a statistically significant increase in the proportion of tumours displaying aggressive features (65.7% of the nodules) when compared to Group 2 which demonstrated aggressive features in 21.7% of the nodules, and Group 3 which demonstrated aggressive features in 11.1% of the nodules (*p*-value < 0.01) (Fig. 1).

**Fig. 2:**
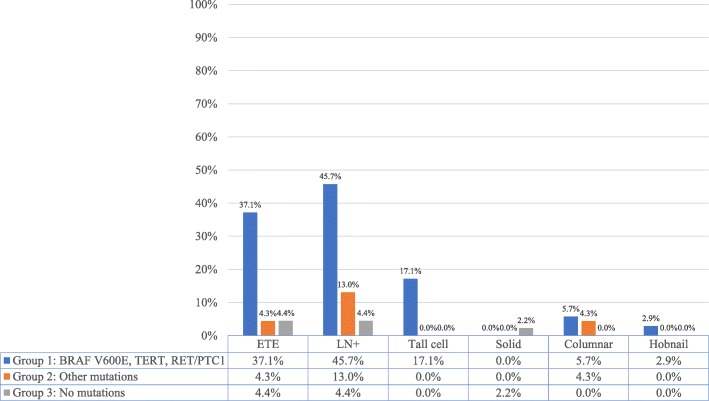
Aggressive features per group (%). Percentage of individual aggressive features in each group was calculated

The tumours in Group 1 demonstrated the following on final pathology: ETE in 11.4% (*n* = 4), micro ETE in 25.7% (*n* = 9), LN+ in 45.7% (*n* = 16), tall cell variant in 17.1% (*n* = 6), columnar cell variant in 5.7% (*n* = 2), and hobnail variant in 2.9% (*n* = 1). In comparison, the tumours in Group 2 demonstrated: micro ETE in 4.3% (*n* = 1), LN+ in 13% (*n* = 3), and columnar cell variant in 4.3% (*n* = 1). The tumours in Group 3 demonstrated: micro ETE in 4.4% (*n* = 2), LN+ in 4.4% (*n* = 2), and solid variant in 2.2% (*n* = 1) (Fig. 2).

### Aggressive features for specific mutations

**Fig. 3:**
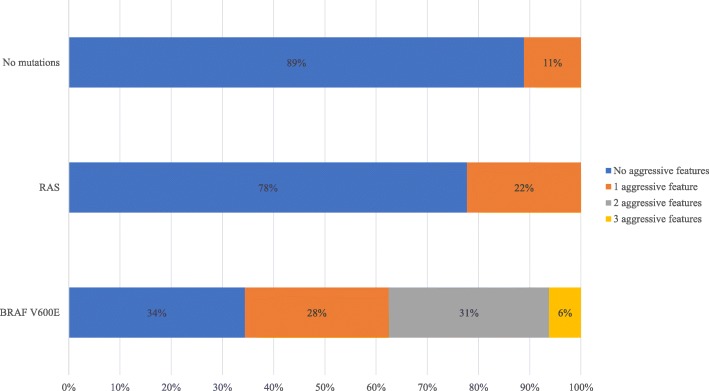
Number of aggressive features per mutation (%). Tumours were stratified in groups that either demonstrated no aggressive features, 1, 2, or 3 aggressive features

The number of aggressive features within each tumour was compared. This demonstrated that *BRAF V600E* tumours were more likely to express multiple aggressive features when compared to *RAS* and tumours where no mutation was detected. *BRAF V600E* was the only mutation in which more than 1 aggressive feature within one tumour was present. These findings are demonstrated in Fig. 3. It is worth noting that the number of tumours exhibiting *BRAF K601E*, *TERT*, *RET/PTC1*, and *PAX8-PPARγ* were too low to establish statistical significance.

**Fig. 4:**
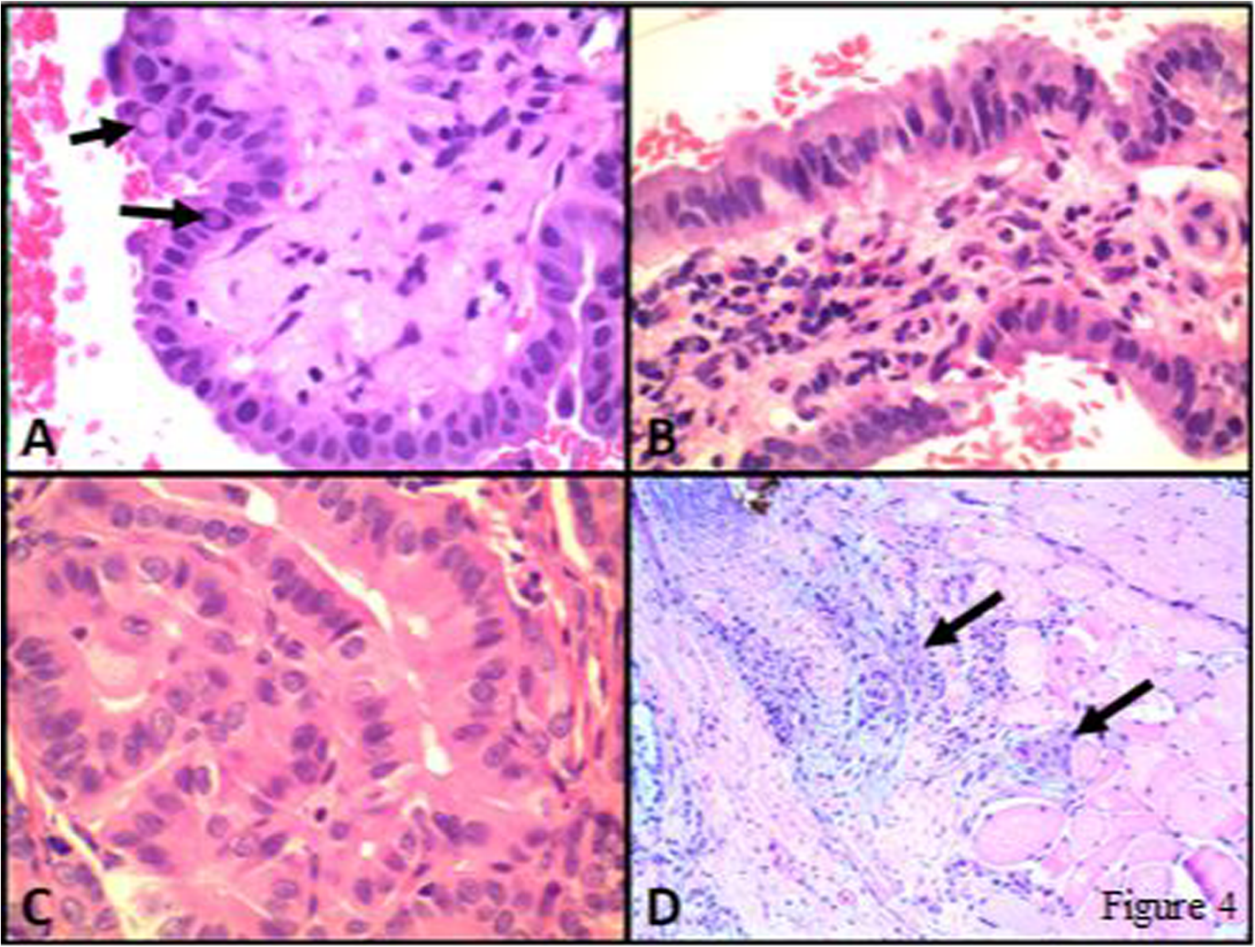
Photomicrographs from a case of PTC, tall cell variant. In this case, tall cell features (i.e. tumour cells 2 or 3 times taller than wide) were present both on the FNA material **a** and **b**, Cell block, H&E), which was classified as Malignant-PTC (Bethesda VI), and on the corresponding resection specimen (**c** and **d**, H&E). The tumour cells also show classical nuclear features of PTC including several intranuclear pseudoinclusions (arrows in A). Extrathyroidal extension was present (D, H&E) with several clusters of tumour cells (arrows) invading skeletal muscle. Molecular testing revealed BRAF V600E mutation

*BRAF V600E* tumours were 5.91 times (relative risk) more likely to demonstrate aggressive features when compared to tumours where no mutation was detected (95% CI 2.49-14.0, *p*-value 0.0001). Supplemental Figure 1 illustrates one of these cases (Fig. 4). Furthermore, *BRAF V600E* tumours were 2.49 times more likely to demonstrate aggressive features when compared to tumours where a *RAS* mutation was detected (95% CI 1.13-5.51, *p*-value 0.024). *RAS* mutations were not found to be statistically more likely to demonstrate aggressive features when compared to tumours with no mutation detected (RR = 2; 95% CI 0.6-6.61, *p* value 0.26). The relative risk of aggressive features is summarized in Table 3.

**Table 3:** Relative Risk of Aggressive Features

Mutation	*BRAF V600E* Relative Risk	95% CI	*p*-value
No mutation	5.91	2.49-14.0	0.0001*
*RAS*	2.49	1.13-5.51	0.024*

*Statistically significant p-value < 0.05

## Discussion

Molecular testing is a diagnostic tool that is used to better understand the nature of thyroid nodules pre-operatively. Much of the literature has focused on molecular testing’s ability to determine whether a patient with an indeterminate thyroid nodule requires surgery [2, 3, 20–23]. Our study’s aim was to take the analysis one step further and to determine whether molecular testing can help with decision-making on the optimal surgical treatment, including the extent and timing of surgery for patients with a thyroid nodule that was suspicious or malignant on thyroid FNA.

Total thyroidectomy is associated with a higher risk of surgical complications and requires patients to take lifelong hormone replacement therapy. Patients undergoing hemithyroidectomy require completion thyroidectomy at times [24]. Therefore, selecting the appropriate initial surgical procedure improves patient care and saves the health care system valuable resources.

In this study, *BRAF V600E, RET/PTC1* rearrangement*,* and *TERT* promoter mutations were found to be associated with aggressive cancers 65.7% of the time. In comparison, 21.7% of nodules that preoperatively tested positive for *BRAF K601E*, *HRAS*, *NRAS*, *KRAS*, or *PAX8-PPARγ* rearrangement mutations and 11.1% of nodules where no mutation was detected were found to have aggressive features in the postoperative pathology report (*n* = 103, *p* < 0.01). Similar findings in microcarcinomas have recently been reported [25]. This finding demonstrates the clinical validity of molecular testing as outlined by the 2015 American Thyroid Association Guidelines by successfully distinguishing different groups of patients based on expected disease outcome (likelihood of recurrence). In addition to guiding the decision of the extent of surgery, the association of certain mutations with aggressivity may allow the test to guide the timing of surgery.

There are several limitations in this study, including the inherent limitations of a retrospective study. As a single center study in Montreal, Canada, a geographic selection bias was introduced. Furthermore, since molecular testing is not available at most Canadian centers, a selection bias for patients who had access to the test was also introduced. Also, the molecular test that was used is a limited panel, as a result, there are mutations and genetic alterations that are not being detected. Finally, surgeons and pathologists were not blinded to the results of molecular testing. Future studies should involve a more comprehensive molecular testing panel, a double blinded and multicenter approach, with financial aid, to minimize these limitations.

## Conclusions

In this study, *BRAF V600E, RETPTC1 rearrangement,* and *TERT* promoter mutations were found to be associated with more aggressive cancers when compared to malignancies without one of these mutations. This association demonstrates that molecular testing may be a valuable pre-operative tool to help guide the decision as to the extent of surgery, hemi-thyroidectomy versus total thyroidectomy and possible central compartment neck dissection, although further prospective studies with larger sample sizes are needed. Choosing the optimal extent of surgery will lead to improved patient care and save the health care system valuable resources.

## Supplementary information

**Additional file 1.** Krasner et al. raw data bank.

## Data Availability

The dataset supporting the conclusions of this article is included within the article (and its additional files.)

## Abbreviations

ETE: Extra-Thyroidal Extension
FNA: Fine Needle Aspiration
LN +: Lymph Node Metastasis
U/S: Ultrasound
